# Genome analysis of a halophilic bacterium *Halomonas malpeensis* YU-PRIM-29^T^ reveals its exopolysaccharide and pigment producing capabilities

**DOI:** 10.1038/s41598-021-81395-1

**Published:** 2021-01-18

**Authors:** Athmika, Sudeep D. Ghate, A. B. Arun, Sneha S. Rao, S. T. Arun Kumar, Mrudula Kinarulla Kandiyil, Kanekar Saptami, P. D. Rekha

**Affiliations:** Yenepoya Research Centre, Yenepoya (Deemed to be University), University Road, Deralakatte, Mangalore, 575018 India

**Keywords:** Biotechnology, Microbiology

## Abstract

*Halomonas malpeensis* strain YU-PRIM-29^T^ is a yellow pigmented, exopolysaccharide (EPS) producing halophilic bacterium isolated from the coastal region. To understand the biosynthesis pathways involved in the EPS and pigment production, whole genome analysis was performed. The complete genome sequencing and the de novo assembly were carried out using Illumina sequencing and SPAdes genome assembler (ver 3.11.1) respectively followed by detailed genome annotation. The genome consists of 3,607,821 bp distributed in 18 contigs with 3337 protein coding genes and 53% of the annotated CDS are having putative functions. Gene annotation disclosed the presence of genes involved in ABC transporter-dependent pathway of EPS biosynthesis. As the ABC transporter-dependent pathway is also implicated in the capsular polysaccharide (CPS) biosynthesis, we employed extraction protocols for both EPS (from the culture supernatants) and CPS (from the cells) and found that the secreted polysaccharide i.e., EPS was predominant. The EPS showed good emulsifying activities against the petroleum hydrocarbons and its production was dependent on the carbon source supplied. The genome analysis also revealed genes involved in industrially important metabolites such as zeaxanthin pigment, ectoine and polyhydroxyalkanoate (PHA) biosynthesis. To confirm the genome data, we extracted these metabolites from the cultures and successfully identified them. The pigment extracted from the cells showed the distinct UV–Vis spectra having characteristic absorption peak of zeaxanthin (λ_max_ 448 nm) with potent antioxidant activities. The ability of *H. malpeensis* strain YU-PRIM-29^T^ to produce important biomolecules makes it an industrially important bacterium.

## Introduction

*Halomonas malpeensis* YU-PRIM-29^T^ belongs to Halomonadaceae family within the Gammaproteobacteria. The species of the genus *Halomonas* are Gram-negative, rod-shaped, aerobic and non-spore forming bacteria^1^. They are highly halotolerant (up to 20% salinity) and are mostly associated with saline environments^2^. Many members have been isolated from diverse saline or hyper-saline environments such as ocean water^3^ and hyper-saline lakes^4^ and also reported from varying pH and temperature conditions^5^.

Bacterial survival in the challenging extreme habitats is possible due to its unique capabilities in the biosynthesis of metabolites that offer protection against such conditions. These molecules serve as osmolytes and protect the cells from damage while allowing normal cellular functions. Many members of the genus *Halomonas* also produce pigments and exopolysaccharides (EPS)^5^ having specific functional role in the adaptation and survival in the extreme environmental conditions^6^. In addition to providing protection against the osmotic stress prevailing in the marine environments, the EPS serves as a tactic for adhesion to solid surfaces and helps in the retention of water and nutrients. It imparts stability to the structure of biofilms and forms a layer surrounding the cell to provide an effective barrier against salinity, bacterial attacks and facilitate biochemical interactions among the cells and the adjacent environment^7,8^.

The important members of halophilic bacteria that produce commercially important EPS from Halomonadaceae family include *Volcaniella eurihalina*, *Deleya marina, H. maura*, *H. anticariensis*, *H. ventosae*, *H. almeriensis*, *H. nitroreducens*, *H. cerina*, *H. fontilapidosi*, *H. rifensis*, and *H. stenophila*^9^. The structural and functional diversity among the EPS are seen depending on the species or the strain of the bacteria. Some of the EPS produced by *Halomonas* spp. possess excellent emulsifying potential. Examples include *H. ventosae* strains Al12^T^ and Al16 and *H. anticariensis* strains FP35^T^ and FP36^10^. The EPS of *H. eurihalina* H96 also exhibits high emulsifying activity and an ability to form a gel in acidic pH^11^. *H. maura* produce an EPS named mauran, containing mannose, galactose, glucose, and glucuronic acid. It forms highly viscous solutions, similar in properties to that of xanthan^12^. These EPS exhibit amphiphilic nature suitable for biodegradation of oils^13^.

EPS biosynthesis is an energy exhaustive process involving three significant steps i.e., synthesis of the nucleoside diphosphate monosaccharides, polymerisation of the repeating unit, its transport and secretion. Intracellular production of the EPS includes substrate uptake, metabolite pathway and the assembly. The internalized sugar molecules are converted to specific monosaccharide by enzymatic chemical modification^14^. Based on the presence of enzymes necessary for addition of chemical groups such as, acetyl, pyruvate, phosphate etc., conversion of the monosaccharide occurs. These modified monosaccharides are then converted to nucleoside diphosphate sugars and are assembled on undecaprenyl pyrophosphate that is found attached to the inner plasma membrane^15^. Polymerisation occurs in the inner membrane by any of the two mechanisms: Wzx/Wzy-dependent pathway and ABC transporter-dependent pathway^16^. The role of the ABC transporter-dependent pathway is well established in the capsular polysaccharide (CPS) production; however, its involvement in the EPS production is also reported^17^.

In addition to the production of osmolytes, *H. malpeensis* produces a yellow pigment that may be of industrial importance. Microbially derived natural pigments have advantages as the production is not limited by season coupled with lower costs for downstream processing^18,19^. The natural pigments have high industrial value due to their enormous applications as antioxidants, functional foods, natural food colorants, antimicrobial agents, etc. Among the characterised pigments of *Halomonas* species, *H. elongata* and *H. aquamarina* are known to produce β-carotene and bacterioruberin respectively^20–22^. Among the many other metabolites of *Halomonas* species ectoine has attracted a great interest as an osmolyte having application in cosmetic industry^23,24^.

The whole genome analysis of a few members of *Halomonas* species has revealed the versatile functional capabilities of the bacteria^25–27^. However, in-depth analysis of the EPS biosynthesis pathway in *Halomonas* species is not yet elucidated. Hence, the genome analysis of *H. malpeensis* was performed to understand the EPS biosynthesis and export pathways as well as to provide necessary information on other industrially exploitable metabolites.

## Results

### General features of the *H. malpeensis* genome

**Figure 1:**
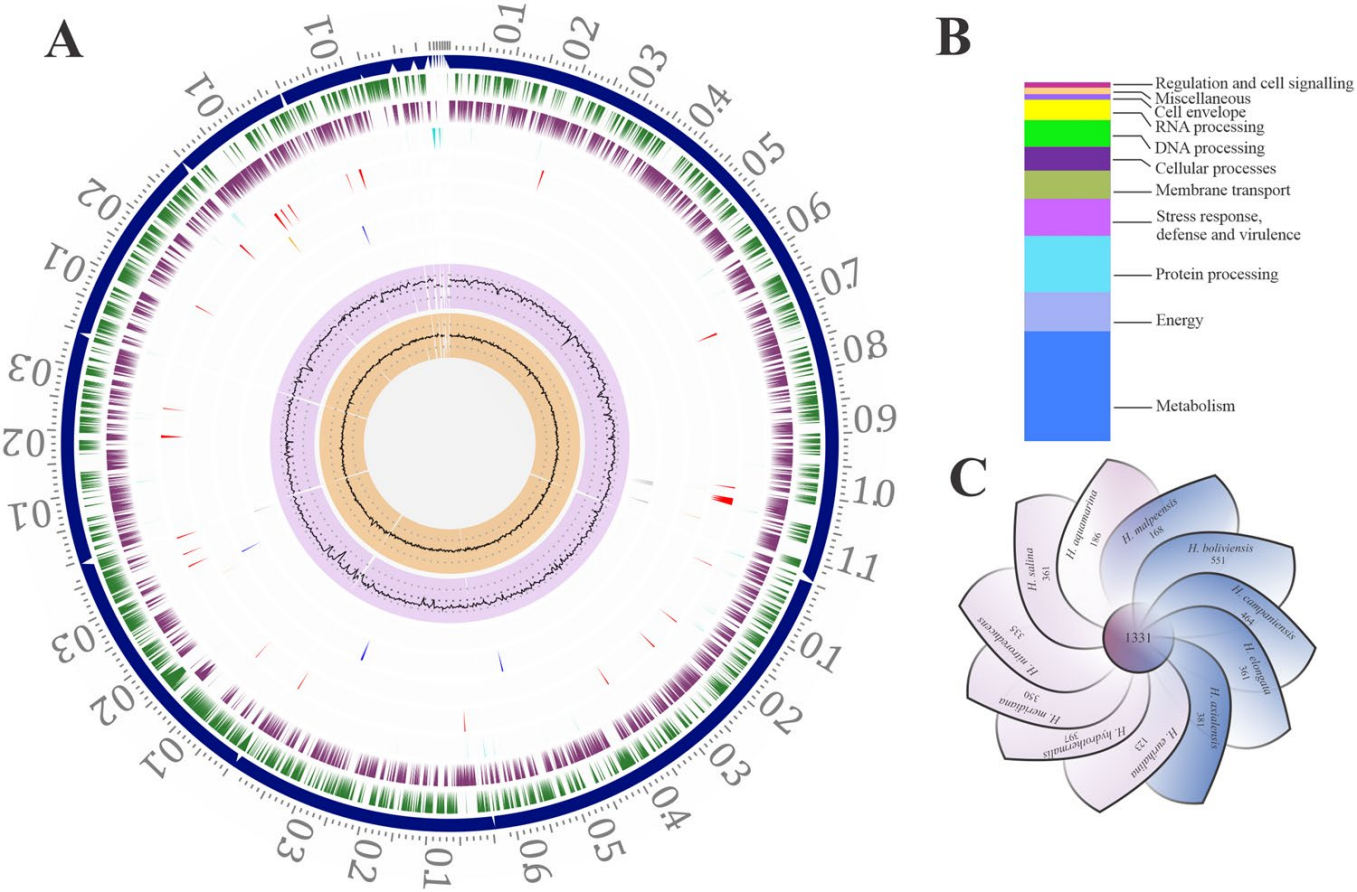
Genomic features and a comparative genomic analysis of *H. malpeensis* YU-PRIM-29^T^. (**A**) Circular plot representing the genome annotations of *H. malpeensis* YU-PRIM-29^T^. Circles are numbered from 1 (outermost) to 8 (innermost). Circle 1 represents the contigs; Circles 2 and 3 show the locations of predicted coding sequences (CDSs) on the forward and reverse strands, respectively; Circle 4, RNA genes; Circle 5, CDS with homology to known antimicrobial resistance genes; Circle 6, CDS with homology to known virulence factors; Circle 7, % G + C; Circle 8, GC skew [(G − C)/(G + C)]. (**B**) Bar-chart representing PATRIC subsystems in the *H. malpeensis* YU-PRIM-29^T^ genome. (**C**) Genomic diversity of 11 *Halomonas* strains. Each strain is represented by a petal. The central number represents the orthologous CDSs present in all strains. Overlapping regions show the number of CDSs conserved only within the specified genomes, while the number of CDSs unique to each strain is represented in non-overlapping portions. The total number of CDSs within each genome is enumerated beneath the strain name.

The complete genome sequencing of *H. malpeensis* YU-PRIM-29^T^ performed using Illumina sequencing produced 1,173,355 paired end raw reads. The genome consists of 3,607,821 bp distributed in 18 contigs with G + C content of 63.8% (Fig. 1A). It contains 3337 protein coding genes (CDS) and 53% of the annotated CDS having putative functions, while the remaining 47% genes are annotated as hypothetical proteins (Fig. 1B). The salient features of the genome are summarized in Table 1. A total of 251 pathways were identified and the important metabolic pathways in the genome are glycolysis, gluconeogenesis, glyoxylate bypass, TCA cycle and pentose phosphate pathway. The biosynthesis pathway for amino acids includes serine, threonine, cysteine, methionine, histidine, arginine, proline, valine, leucine, isoleucine, phenylalanine, tyrosine, tryptophan and lysine. The metabolic pathways for vitamins like biotin, thiamine and riboflavin are also assigned.

**Table 1:** General features of the *H. malpeensis* YU-PRIM-29^T^ draft genome.

Feature	*H. malpeensis* YU-PRIM-29^T^
Domain	Bacteria
Taxonomy	Proteobacteria; Gammaproteobacteria; Oceanospirillales; Halomonadaceae; *Halomonas*
Genome size	3,607,821 bp
G + C content	63.75%
Completeness	99.00%
Contamination	0.00%
Number of coding sequences (CDSs) in PATRIC	3337
Proteins with functional assignments	2653
Hypothetical proteins	684
Proteins with EC number assignments	930
Proteins with KEGG pathway assignments	700
Genes assigned to COGs	1817
Number of tRNA	58
Number of rRNA	12
G + C content of tRNA	58.51%
G + C content of rRNA	56.57%
N50 value	410,886 bp
L50 value	3

The distribution of genes into clusters of orthologous groups (COGs) functional categories is listed in Table 2. Using PATRIC Protein Family Sorter tool, a core genome containing 1,331 protein-coding genes that are shared across 11 *Halomonas* strains (Supplementary Table S1) was identified as shown in Fig. 1C. This core genome is made up of only 22–42% of the proteome of each strain, signifying a high amount of genomic diversity among species of the *Halomonas* genus. The pan genome and core-genome contain 8,011 and 1,331 genes respectively.

**Table 2:** Number of genes associated with the general cluster of orthologous group (COG) functional categories in *H. malpeensis* YU-PRIM-29^T^ genome.

COG code	Number of genes	Percentage	Description
**Cellular processes and signalling**			
D	30	0.90	Cell cycle control, cell division, chromosome partitioning
M	121	3.63	Cell wall/membrane/envelope biogenesis
N	61	1.83	Cell motility
O	81	2.43	Post-translational modification, protein turnover, chaperones
T	67	2.01	Signal transduction mechanisms
U	34	1.02	Intracellular trafficking, secretion, and vesicular transport
V	29	0.87	Defense mechanisms
W	0	0.00	Extracellular structures
Y	0	0.00	Nuclear structure
Z	0	0.00	Cytoskeleton
**Information storage and processing**			
A	1	0.03	RNA processing and modification
B	1	0.03	Chromatin structure and dynamics
J	193	5.78	Translation, ribosomal structure and biogenesis
K	62	1.86	Transcription
L	85	2.55	Replication, recombination and repair
X	17	0.51	Mobilome: prophages, transposons
**Metabolism**			
C	127	3.81	Energy production and conversion
E	179	5.36	Amino acid transport and metabolism
F	68	2.04	Nucleotide transport and metabolism
G	89	2.67	Carbohydrate transport and metabolism
H	104	3.12	Coenzyme transport and metabolism
I	64	1.92	Lipid transport and metabolism
P	107	3.21	Inorganic ion transport and metabolism
Q	30	0.90	Secondary metabolites biosynthesis, transport and catabolism
**Poorly characterised**			
R	120	3.60	General function prediction only
S	147	4.41	Function unknown
–	1520	45.55	Not in COGs

### EPS biosynthesis, transport of sugars and nucleotide sugar synthesis

The key EPS biosynthesis pathway was identified by employing Kyoto Encyclopedia of Genes and Genomes (KEGG) and KEGG Automatic Annotation Server (KAAS) functional annotation tools along with Prokka and Pathosystems Resource Integration Center (PATRIC) annotations. The genes identified from the whole genome sequence were verified by performing a translated Basic Local Alignment Search Tool (BLAST) against known sequences available in National Center for Biotechnology Information (NCBI) Genbank database (https://www.ncbi.nlm.nih.gov/genbank/). In general, based on the available literature, the bacterial EPS synthesis follows one of the three mechanisms: Wzx/Wzy-dependent pathway, ABC transporter-dependent pathway and/or synthase dependent pathway^28^. However, the ABC transporter-dependent pathway is mostly associated with CPS biosynthesis. Annotation of the EPS biosynthesis pathway in *H. malpeensis* shows the presence of 184 ABC transporters, but the genes involved in Wzx/Wzy-dependent (*wzx*, *wzy* and *wzz* genes) and synthase dependent pathways are absent (Supplementary Table S2). The EPS synthesis machinery involves three significant steps i.e., synthesis of the nucleoside diphosphate sugars (NDP sugars), polymerisation of the repeating units followed by translocation and secretion. Further, the EPS gene cluster was identified with antiSMASH (Node 477,025–155,069 bps) and showed 10% similarity with *Lactobacillus johnosonii* EPS cluster^29^. However, the database is limited as only a few biosynthetic gene clusters (BGC) of EPS are present in the MIBiG database with most of them belonging to Gram-positive bacteria^30^.

**Figure 2:**
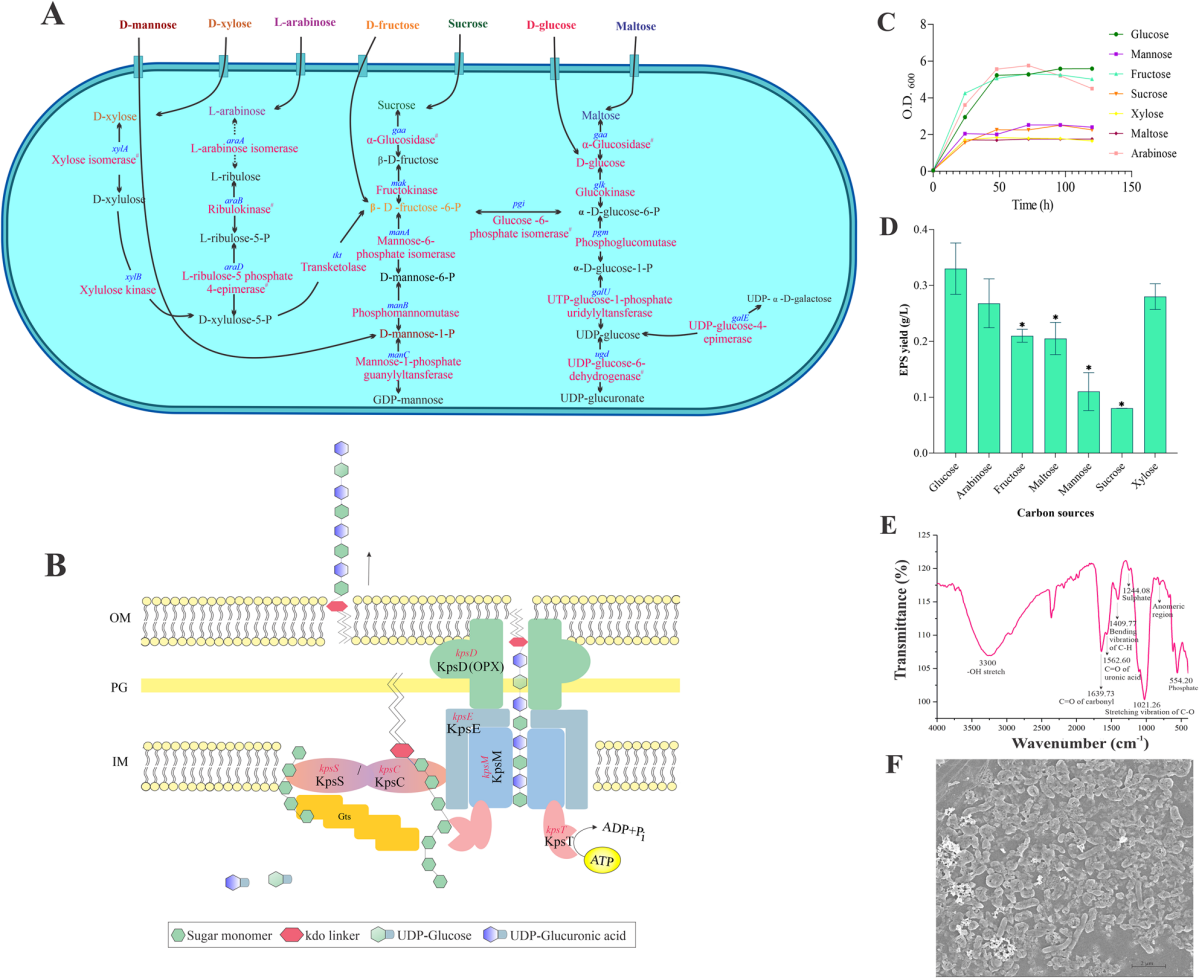
(**A**) Genes identified for the polysaccharide biosynthesis in *H. malpeensis* YU-PRIM-29^T^ from the genomic data. ^#^Results obtained from tBLASTn analysis and the details are provided in the Supplementary Tables S2 and S10. (**B**) ABC transporter-dependent assembly and transport of polysaccharides in *H. malpeensis*. (**C**) Influence of carbon source on growth of *H. malpeensis*. Data points are OD_600_ values obtained from broth cultures incubated with different carbon sources recorded at different time points. (**D**) EPS yield obtained from different carbon sources at 48 h, *indicates p value < 0.001. Data points are mean ± SD and n = 3. (**E**) FTIR spectra of EPS with peaks showing the important bands and (**F**) FESEM image of biofilm formed by *H. malpeensis* on glass surface.

In ABC transporter-dependent pathway, the polysaccharide is exported across the inner membrane through various transporters. The transporters that are involved in the transport of molecules are listed in Table 3. A total of 184 genes in the ATP binding cassette (ABC) transporters are identified for the transport of substrates like ions, salts, sugars, vitamins, amino acids and purines across the cell membrane in an ATP-dependent manner. Apart from ABC transporters *H. malpeensis* also possesses genes for carbohydrate transport involved in the uptake of D-mannose, D-xylose, L-arabinose, D-fructose, sucrose, D-glucose and maltose (Supplementary Table S3). On its import into the cell’s interior, the sugars are converted to nucleotide sugar precursors. Based on the functional annotation, the enzymes involved in the synthesis of UDP-glucose and GDP-mannose from monosaccharides are shown in Fig. 2A. The gene *galU* coding for the enzyme UTP-glucose-1-phosphate uridylyl transferase (EC 2.7.7.9), *galE* for UDP-glucose-4-epimerase (EC 5.1.3.2) involved in the production of UDP-galactose, *ugd* for UDP-glucose-6-dehydrogenase (EC 1.1.1.22) for the production of UDP-glucuronate from UDP-glucose and the gene *manC* for mannose-1-phosphate guanylyl transferases (EC 2.7.7.13) for GDP-mannose biosynthesis are identified (Supplementary Table S2). In the biosynthesis process, the sugar polymerisation is initiated by the action of glycosyltransferases by mediating glycosidic bond formation and these enzymes use sugar donors that contain nucleoside phosphate or lipid phosphate leaving groups. The presence of 29 glycosyltransferases is identified using PATRIC, Rapid Annotations using Subsystems Technology (RAST), dbCAN2 and Prokka annotation of which four are found in the EPS cluster hinting their role in transport of the produced EPS. The detailed list of the glycosyltransferases is given in Table 4.

**Table 3:** Transporter systems for transfer of molecules identified in the *H. malpeensis* YU-PRIM-29^T^ genome*.*

Category	Molecules
Metal ions	Na, K, Cu, Mg, Co, Ni, Pb, Cd, Zn, Hg, Fe, F, Ca, Mn
Anions	Phosphate, chromate, phosphite, nitrate, nitrite, sulphate, aminobenzoyl-glutamate
Other cations	Ammonium
Amino acids	Serine, threonine, proline, choline, histidine/lysine/arginine/ornithine, cysteine, methionine, glutamine, glutamate
Carbohydrates	Ribose/xylose/arabinose/galactose, glucose, maltose, glyceraldehyde-3-phosphate, fructose
Other molecules	Vitamin B12, dicarboxylate, tricarboxylate, glycine betaine, riboflavin, spermidine, putrescine, glycerol-3 phosphate, ectoine, hydroxyectoine, queuosine precursor, 2-nitroimidazole, sialic acid

**Table 4:** Glycosyltransferases present in *H. malpeensis* YU-PRIM-29^T^ genome.

PATRIC ID	Encoded protein	Length (aa)	Molecular weight (kDa)	GT family
fig|2745.436.peg.139	Glycosyltransferase, group 1	373	41.13	GT4
fig|2745.436.peg.211	Glycosyltransferase, group 1	641	69.25	GT4
fig|2745.436.peg.583	Zeaxanthin glycosyltransferase	429	46.36	
fig|2745.436.peg.676	Glycosyltransferase	1023	117.2	GT4
fig|2745.436.peg.886	Glycosyltransferase, group 1	351	38.37	
fig|2745.436.peg.887	Glycosyltransferase, group 1	698	76.28	GT4
fig|2745.436.peg.888	Glycosyltransferase, group 1	291	32.79	GT4
fig|2745.436.peg.906	Glycosyltransferase	387	42.3	GT4
fig|2745.436.peg.1361	peptidoglycan transglycosylase	238	27.17	GT51
fig|2745.436.peg.1426	ADP-glucose transglucosylase	565	61.41	GT5
fig|2745.436.peg.1489	Multimodular transpeptidase-transglycosylase	842	92.15	GT51
fig|2745.436.peg.1748	Glycosyltransferase, group 2 family	316	36.12	
fig|2745.436.peg.2024	Glycosyltransferase WecB/TagA/CpsF	225	25.94	GT26
fig|2745.436.peg.2035	Glucosyl-3-phosphoglycerate synthase	406	46.26	GT81
fig|2745.436.peg.2038	Uncharacterised glycosyltransferase YcjM	606	67.74	GH13
**fig|2745.436.peg.2094**	**Glycosyltransferase, group 2**	**657**	**76.92**	
**fig|2745.436.peg.2104**	**Glycosyltransferase, group 1**	**992**	**108.63**	
**fig|2745.436.peg.2106**	**Glycosyltransferase, group 2 family**	**727**	**82.2**	**GT2**
**fig|2745.436.peg.2107**	**Glycosyltransferase, group 1**	**256**	**39.47**	
fig|2745.436.peg.2292	Peptidoglycan glycosyltransferase FtsW (EC 2.4.1.129)	393	42.95	
fig|2745.436.peg.2293	N-acetylglucosaminyltransferase	365	38.94	GT28
fig|2745.436.peg.2551	Glycosyltransferase, group 1			
fig|2745.436.peg.2881	Biofilm PGA synthesis N-glycosyltransferase	424	48.67	GT2
fig|2745.436.peg.3032	Lipid-A-disaccharide synthase	390	42.57	GT19
fig|2745.436.peg.3208	ADP-heptose–lipooligosaccharide heptosyltransferase II	350	37.89	GT9
fig|2745.436.peg.3210	Lipopolysaccharide biosynthesis glycosyltransferase	355	40.53	
fig|2745.436.peg.3212	glycosyltransferase, group 1	372	40.67	GT4
fig|2745.436.peg.3211	ADP-heptose–lipooligosaccharide heptosyltransferase II	345	36.82	GT9
fig|2745.436.peg.3213	3-deoxy-D-manno-octulosonic acid kinase	238	26.14	GT9

The GT’s in bold are the glycosyl transferases identified in the EPS gene cluster.

For the export of EPS outside the cell, *H. malpeensis* is proposed to follow an ABC transporter-dependent pathway^16^. The genes coding for Kps proteins involved in ABC transporter-dependent pathway such as *kpsD*, *kpsM*, *kpsE*, *kpsT, kpsS* and *kpsC* are identified in the genome. The *kps* gene cluster has been predicted using antiSMASH. The structural details of these proteins were searched using InterPro database. The KpsS and KpsC proteins (β-Kdo transferases) synthesise a capsular polysaccharide export system protein (oligo-Kdo linker); KpsT is an ATP binding protein; KpsM is an ABC transporter permease protein that interacts with KpsT; KpsD is an export system periplasmic protein and KpsE is an export system inner membrane protein (Fig. 2B).

The possible genes involved in EPS modifications were searched using Prokka annotation and tBLASTn. A gene *ugd* coding for UDP-glucose-6-dehydrogenase (EC 1.1.1.22) involved in the conversion of the nucleotide sugar UDP-glucose to UDP-glucuronic acid is found.

As the ABC transporter-dependent pathway is mainly associated with CPS, we followed extraction procedures for both CPS and EPS and observed that EPS was predominantly extracted. Ability of the bacteria to produce EPS was tested using different carbon source supplementation. Among the carbon sources tested based on the genomic data, the growth was favoured by all the sugars (Fig. 2C), while, the EPS yield was higher in D-glucose, D-xylose, D-fructose and L-arabinose supplemented media (Fig. 2D). The EPS yield in the media containing D-mannose and maltose were significantly (p < 0.001) lower compared to the others. Biochemical analysis of the EPS showed 76% total sugar, 5% protein on w/w basis and among the sugars more than 50% were uronic acid containing. The Fourier Transform Infrared Spectroscopy (FTIR) analysis showed the characteristic carbohydrate peaks at 3600–2900 cm^−1^ (–OH groups), 2937 cm^−1^ (–CH_2_ stretching), 1735 cm^−1^ (uronic acid) and 1045 cm^−1^ (acetyl group) among others (Fig. 2E).

The extracted EPS showed emulsification activity against petroleum hydrocarbons. The highest emulsification index (EI_24_) was against toluene 64 ± 4%, followed by kerosene 63 ± 4%, xylene 62 ± 4%, hexane 60 ± 6% and petrol 53 ± 2%. These values were significantly higher than Tween 20 (used as positive control) at the same concentration.

### Chemotaxis and biofilm formation

Chemotaxis is one of the mechanisms adapted by the bacteria to sense the external environment in order to modify the mode of growth. The chemoattractant molecules influence the flagellar motor to direct the movement of the bacterial cell either towards or away from the chemical signal. The genes coding for the proteins belonging to the chemosensory pathway such as *cheR, cheB, cheA, cheW, cheY, cheZ, fliG, fliM, fliN, motA* and *motB* in the genome of *H. malpeensis* were identified using KEGG. The membrane cofactor protein (MCP; CD46) can allow the attractant or repellent to be taken into the cell. The function of MCP is regulated by *cheR* that codes for methyl transferase (EC 2.1.1.80) and *cheB* coding for methyl esterase (EC 3.1.1.61) which in turn are regulated by *cheA* gene encoding for two component signalling kinase (EC 2.7.13.3) and *cheW* for a coupling protein. The CheA and CheW proteins activate CheY to regulate the function of MotA and MotB through activation of the *fli* genes (*fliG*, *fliM* and *fliN*). The genes for the biofilm formation such as, *wspA*, *wspE*, *wspF*, *wspR*, *sadC*, *tpbB*, *mucR* and *algA* were identified by KEGG (Supplementary Table S4).

To test whether the bacteria form biofilm, the crystal violet staining and Field Emission Scanning Electron Microscopy (FESEM) were used (Fig. 2F). The bacteria formed strongly adherent biofilms and the intensity increased over the incubation period as recorded by the crystal violet staining method. The biofilm intensities corresponded to an OD_580_ value of 1.48, 1.97 and 3.31 at 24 h, 48 h and 96 h of incubation respectively.

### Pigment biosynthesis

**Figure 3:**
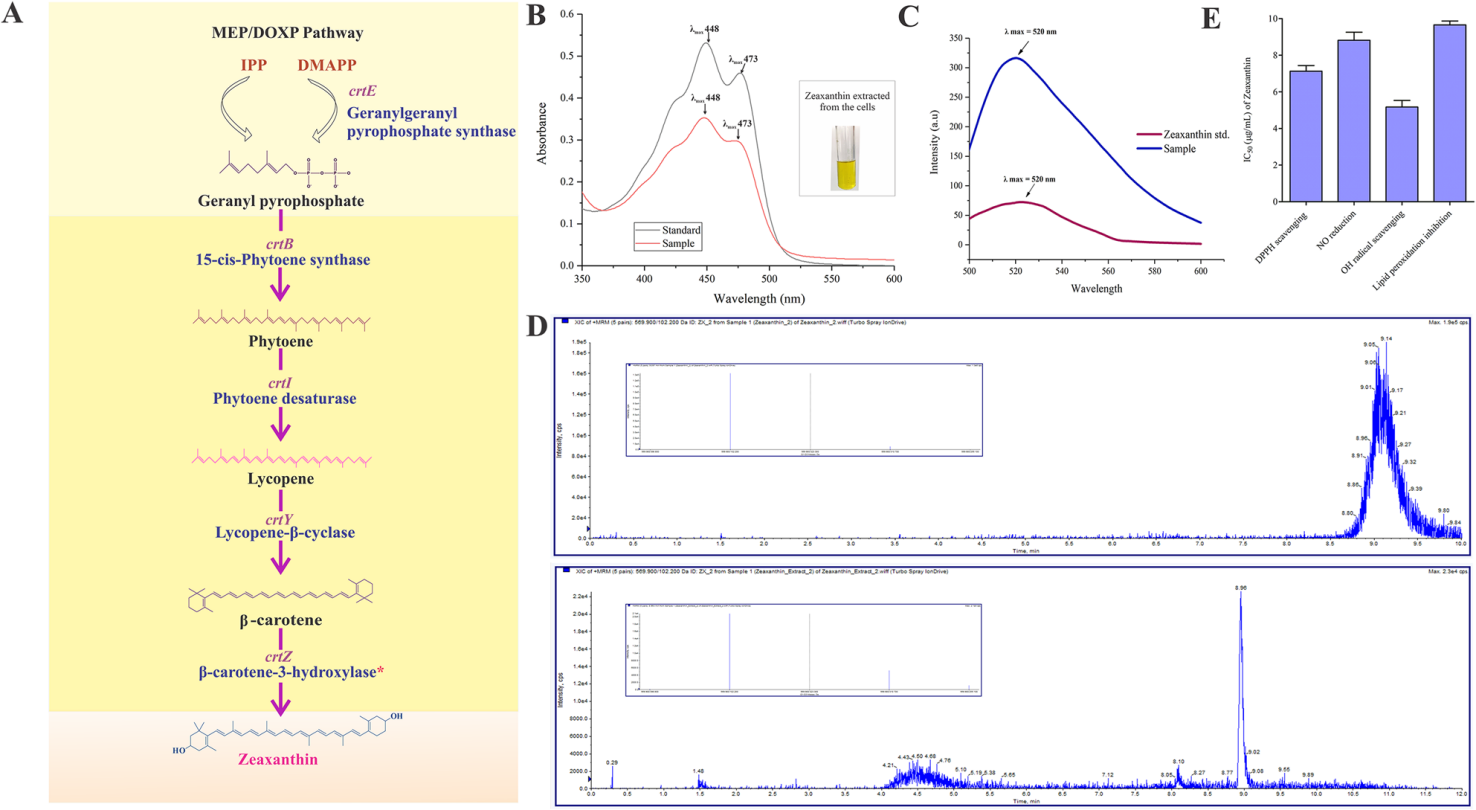
Zeaxanthin biosynthesis in *H. malpeensis* YU-PRIM-29^T^. (**A**) Complete zeaxanthin biosynthesis pathway as predicted from genomic sequence data. The biosynthesis pathway was constructed based on the KEGG database, PATRIC genome annotation and Prokka. *Result obtained from tBLASTn analysis and details are provided in the Supplementary Tables S5 and S10. (**B**) UV–Vis spectra of zeaxanthin extracted from *H. malpeensis.* Standard zeaxanthin was used for comparison. Inset showing solvent extracted zeaxanthin from the cells. (**C**) Fluorescence spectra of zeaxanthin extracted from *H. malpeensis* compared to standard zeaxanthin showing maximum absorption at 520 nm. (**D**) Targeted LC–MS MRM spectra of extracted zeaxanthin with the standard acquired in positive polarity. Two transitions were observed in the extract. (**E**) IC_50_ values (µg mL^−1^) for zeaxanthin showing DPPH scavenging, NO reduction, OH radical scavenging and lipid peroxidation inhibition activities. The bars represent mean ± SD of triplicate results.

Microbial pigments occur in two forms; either secreted by the cell or cell bound. In this bacterium the intense yellow pigment is cell bound and was water insoluble. Hence, it required extraction using organic solvents. The genes *crtB, crtI, crtY* and *crtZ* responsible for the carotenoid biosynthesis were present. These genes are responsible for the enzymes of 15-cis-phytoene synthase (EC 2.5.1.32) that converts geranyl pyrophosphate to phytoene, phytoene desaturase (EC 1.3.99.31), converts phytoene to lycopene, lycopene β-cyclase (EC 5.5.1.19) converts lycopene to β-carotene and β-carotene-3-hydroxylase (EC 1.14.15.24) converts β-carotene to zeaxanthin. The detailed biosynthesis pathway identified for the zeaxanthin production is given in Fig. 3A. Further, the yellow pigment produced by the bacterium was extracted from the cell pellets. It was insoluble in water and was extractable in organic solvent methanol. The visible spectra of the extracted yellow pigment showed characteristic absorption peaks with λ_max_ 448 nm and a shoulder peak at 429 nm corresponding to that of zeaxanthin used as a standard^31,32^ (Fig. 3B). The fluorescence spectral characteristics also corresponded to the zeaxanthin pigment (Fig. 3C). The LC–MS/MS data obtained against the standard confirms the presence of zeaxanthin (Fig. 3D). The isolated pigment showed potent antioxidant activities with IC_50_ values of 7.13 µg, 8.82 µg, 5.17 µg and 9.66 µg for DPPH scavenging, nitric oxide reduction, hydroxyl radical scavenging and lipid peroxidation inhibition respectively (Fig. 3E).

KEGG annotation further revealed the enzymes involved in other pigment production pathways namely betalain pathway and riboflavin pathway. The betalain biosynthesis pathway begins with L-tyrosine that is converted to L-DOPA, which in the presence of 4,5-DOPA dioxygenase extradiol (EC 1.13.11.-) is converted to betalamic acid. This conjugates with amino acids spontaneously and forms yellow betaxanthins. The 4,5-DOPA dioxygenase extradiol gene is involved in this pathway. Riboflavin is a yellow coloured water-soluble pigment that is produced by a wide variety of microorganisms and its biosynthesis in *H. malpeensis* was identified using KEGG. The important proteins in the pathway such as RibA (EC 3.5.4.25), RibD1 (EC 3.5.4.26), RibD2 (EC 1.1.1.193), YigB (EC 3.1.3.104), RibH (EC 2.5.1.78), RibE (EC 2.5.1.9), RibF (EC 2.7.1.26), FAD1 (EC 2.7.7.2), RibB (EC 4.1.99.12) and SsuE (EC 1.5.1.38) were annotated using the genome data. The pathway begins with purine metabolism with seven enzymes namely; GTP cyclohydrolase II (RibA), pyrimidine deaminase (RibD1), pyrimidine reductase (RibD2), uracil phosphatase (YigB), 3,4-DHBP synthase (RibH) and ribityllumazine synthase (RibE) for the formation of the pigment (Supplementary Table S5). Apart from these enzymes, flavin mononucleotide (FMN) hydrolase (EC 3.1.3.102) that converts FMN to riboflavin is present.

### Polyhydroxyalkanoates and ectoine

Polyhydroxyalkanoates (PHAs) are important metabolites that are involved in cellular energy storage. The annotation using PATRIC allowed the identification of acetoacetyl-CoA reductase (PhbB; EC 1.1.1.36) and acetyl-CoA acetyltransferase (PhaA; EC 2.3.1.9) involved in PHA production. Although the presence of PHA operon was not observed in the genome, but, the polyhydroxyalkanoate synthase genes (*phaC1* and *phaC2*), polyhydroxyalkanoate depolymerase (*phaZ1, phaZ2,* and *phaZ3)*, polyhydroxyalkanoate synthesis repressor (*phaR*) and phasin (*phaP*) were identified using tBLASTn.

Ectoine is an industrially important osmolyte that is produced by many strains of *Halomonas* spp., to avoid loss of turgor pressure in extreme environments with high osmotic stress conditions prevailing in marine habitats. Using Prokka and PATRIC annotation the genes involved in the ectoine biosynthesis pathway were curated. These included *lysC*, *ectB*, *ectA*, *ectC* and *ectD*. The genes coding for proteins involved in the degradation of the synthesised ectoine present are *doeA, doeB, doeD* and *doeC*.

**Figure 4:**
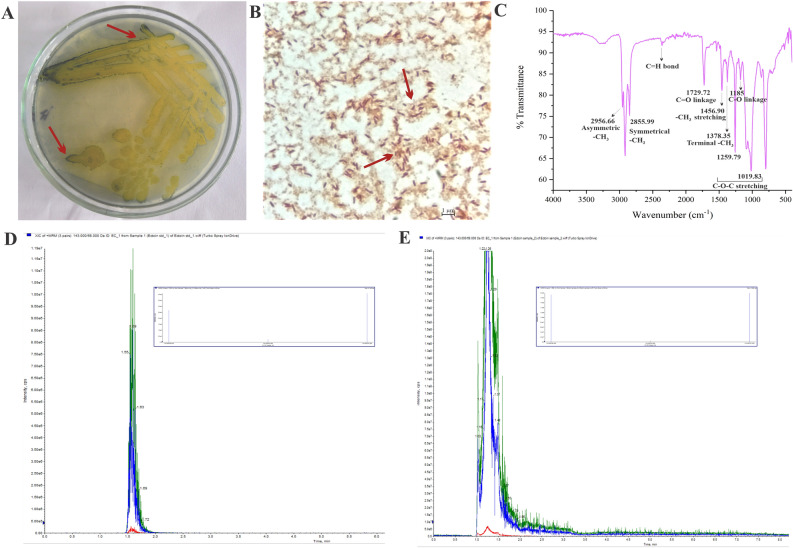
(**A**) Demonstration of polyhydroxyalkanoate production by Sudan Black B staining of *H. malpeensis* YU-PRIM-29^T^*.* Bluish black coloured colonies appearing on plate with 0.02% of dye indicated by red arrow shows the presence of PHA. (**B**) Light microscopy images of Sudan Black B stained cells of *H. malpeensis* observed at 100X. Red arrow indicates the PHA stained with Sudan Black B. (**C**) FTIR spectra of polyhydroxyalkanoate extracted from *H. malpeensis.* (**D**) Targeted LC–MS MRM transition spectra of standard Ectoine. (**E**) Targeted LC–MS MRM transition spectra of ectoine extracted from *H. malpeensis* with fragment peaks shown as inset comparable to the standard acquired in positive polarity.

Experimentally the PHA production was tested by Sudan Black B staining of the colonies and the cells (Fig. 4A,B). We could extract 11.6 mg PHA per gram dry weight of the cell. The IR spectra of the PHA extracted from the cells showed important peaks corresponding to the PHA as follows; C=O groups (1729 cm^−1^), asymmetric methyl group (2956 cm^−1^), symmetrical methyl group (2856 cm^–1^), stretching vibration (1456 cm^−1^) and terminal methyl group vibration (1378 cm^−1^), C–O–C stretching vibration (1260 cm^−1^ and 1020 cm^−1^) and C–O stretching (1185 cm^−1^) (Fig. 4C). Similarly, the ectoine produced by the bacteria was extracted from the cell pellet (~ 0.20 g/L) and was identified using LC–MS/MS against the ectoine standard (Sigma Aldrich, USA) (Fig. 4D).

The genome of *H. malpeensis* YU-PRIM-29^T^ also contains several genes that allow bacterial adaptation to stressful environmental conditions. The important are the spermidine synthase gene (*Srm*) and genes for base excision repair including many DNA glycosylases (Supplementary Table S6). Using antiSMASH 17 putative BGC were identified and these included clusters for saccharide, fatty acids, terpenes, betalactone and ectoine (Supplementary Table S7).

## Discussion

The genus *Halomonas* consists of more than 100 species described from marine and terrestrial high saline environments. Genome annotation helps to identify the functional components in the genome sequence such as genes involved in biosynthesis of important metabolites. From the draft genome of *H. malpeensis* YU-PRIM-29^T^, we have identified the genes involved in the biosynthesis of EPS, PHA, carotenoid (zeaxanthin) and ectoine pathways. The experimental evidences also suggest their production in the bacterial culture.

Bacterial cell surface glycoconjugates are found in various forms either attached to the cell or released into the environment. Many polymers exist between these two states of capsular (cell-bound) or secreted (EPS) slimy forms^33^. The polysaccharide biosynthesis and transport is a complex mechanism that follow one of the following pathways namely; Wzx/Wzy-dependent pathway, ABC transporter-dependent pathway and synthase dependent pathway^34^. While, literature shows that ABC transporter-dependent pathway is mostly associated with CPS production, there are also evidences that implicate its role in EPS biosynthesis^17^. The polysaccharide is exported out of the cell with the help of a translocation pathway which is formed by a polysaccharide copolymerase (PCP) namely, KpsD protein and an outer membrane polysaccharide export protein (OPX) namely KpsE which together form a channel. KpsE determines the length of the polymer chain while KpsD forms the export channel^16,17^. The synthesis and translocation from the periplasm to the cell’s exterior needs the presence of KpsE (PCP-3 family) and KpsD (OPX family) proteins similar to Wza-Wzc proteins^17,35,36^. Four glycosyltransferases involved in the EPS biosynthesis pathway cluster are identified from the draft genome. The presence of genes coding for ABC transporter KpsMT; encoded by *kpsM* and *kpsT* genes^37^ and OPX (KpsD) and PCP (KpsE) proteins in the genome suggests that *H. malpeensis* follows ABC transporter-dependent pathway for the export of polysaccharide and its secretion to the environment as EPS. The genes *kpsS* and *kpsC* are seen in the kps cluster of *H. malpeensis* which are known to code for Kdo linkers mostly associated with CPS^38^. However, there are many reports on EPS production by bacteria with Kdo linkers as reported from *Cobetia*, *Burkholderia* and *Pseudomonas* genus^39–43^.

The growth phase dependent studies on the EPS production in *H. malpeensis* also shows a gradual increase in EPS production during the incubation period peaking at late stationary phase (data not shown). This suggests that, the polysaccharide exported out of the cell may gradually mature and be released to the environment. This may be facilitated by inorganic/chemical mediators present in the environment. Among the reported EPS producing *Halomonas* spp., *H. stenophila* HK30 produces haloglycan type of EPS and in aqueous medium it shows moderate to high viscosity and pseudoplastic behaviour^44^. *H. xianhensis* SUR308 EPS exhibit high viscosity and pseudoplasticity and stable over a wide pH range^45^. The EPS produced from *Halomonas* spp. has demonstrated excellent emulsification activities suitable to remove the oil content in contaminated water and sludge^46^. The EPS extracted from *H. malpeensis* also showed emulsification of petroleum hydrocarbons. The genome based metabolic systems engineering approach in *H. smyrnensis* AAD6^T^ isolate from Camalti Saltern area in Turkey resulted in an increased levan production when compared to the wild type strain^47^.

The biofilm formation capability of *H. malpeensis* was confirmed by the presence of genes involved and experimental results. The biofilm mode of growth provides many advantages to the bacteria such as metabolite exchange platform, better resource capturing, protection from desiccation, drugs, environmental stress and sociomicrobial interaction providing advantages compared to free living/planktonic counterparts^48,49^. The extracellular polymeric substance forms an extracellular matrix for the microbial community in the biofilm and plays a crucial role in binding the cells together^50^. *H. malpeensis* is capable of forming a biofilm on polystyrene material surface as reported in other members of the genus^50,51^. *Halomonas* spp. are also reported as part of corrosive biofilm community in the marine environments^52^. Chemotaxis can also drive biofilm formation based on environmental cues with the expression of *wspA* and *wspE* genes. These genes get activated when the bacterial cells come in contact with the surface that in turn activates *wspR*, *sadC*, *tpbB* and *mucR* genes.

Bacteria produce many pigments through different biosynthesis pathways. For the biosynthesis of carotenoids, lycopene β-cyclase is needed. Phytoene desaturase catalyzes the conversion of phytoene to lycopene by desaturation at four sites^53^. Phytoene synthase catalyzes the condensation reaction of two molecules of geranylgeranyl diphosphate to produce phytoene, a precursor of β-carotene. They produce triterpene and tetraterpene precursors for many diverse sterol and carotenoid end products. *H. malpeensis* contains all the enzymes involved in carotenoid pathway and precursor pathway. Zeaxanthin is an important antioxidant, a product of carotenoid pathway and the enzymes for its synthesis are detected in the genome of *H. malpeensis*. However, *H. elongata* that does not produce zeaxanthin was genetically engineered to produce β-carotene by expressing carotenoid pathway genes *crtE*, *crtY*, *crtI*, and *crtB* derived from *Pantoea agglomerans* and IPP isomerase gene from *Haematococcus pluvialis*^20^. Other pigments produced from the *Halomonas* spp. are, bacterioruberin, a carotenoid derivative from *H. aquamarina* MB598^21^ and aminophenoxazinones from *Halomonas* sp*.* GWS-BW-H8hM^54^.

In addition to carotenoid pathway, the genes involved in the biosynthesis of betalain and riboflavin are identified in *H. malpeensis*. Betalains, the yellow or violet pigments usually synthesised in plants and fungi are reported to be synthesised in bacteria such as *Gluconacetobacter diazotrophicus*. However, it requires a growth medium supplemented with L-DOPA^55^. The genome of *H. malpeensis* contained the gene coding for the enzyme, 4,5-DOPA dioxygenase extradiol.

*Halomonas* spp. are also significant for the production of ectoine, a well-known osmolyte which is produced and released in response to the varying salinity stress. These molecules function as compatible solutes, have no disturbance to the cell even at higher concentrations and reduce the detrimental effect of freezing, desiccation and high temperatures^56^. They do so by interacting with the cell’s protein and contributing to protein folding and are responsible for increased protein stability^57^. Ectoine is now one of the widely used compatible solute in cosmetic industries as skin protectants and anti-ageing products^58^, in healthcare products as anti-inflammatory agents for treatment of allergies and for the treatment of epithelial derived inflammatory ailments^59^. Currently, *H. elongata* is the preferred strain for industrial production of ectoine^2^. *H. malpeensis* possesses all the genes coding for the enzymes and transporters involved in ectoine biosynthesis pathway (Supplementary Table S8). The bacterium was able to produce PHA that confirms the role of genes involved in the PHA biosynthesis pathway. Studies on the distribution of PHA genes in *Halomonas* sp. SF2003 suggest that the genes are not clustered in one operon but distant from each other^60^. A similar scattered occurrence of PHA relevant genes was reported in the genome of *Halomonas* sp. TD0. The *phaP* and *phaC1* are connected with a space of 92 bp similar to our observation showing a gap of 90 bp between the two genes^61^.

In summary, the draft whole genome of halophilic strain *H. malpeensis* YU-PRIM-29^T^ was annotated using bioinformatic tools to explore the production of commercially important metabolites. The EPS, pigment, PHA and ectoine biosynthesis pathways described in this study may provide prospects to exploit this bacterium industrially.

## Methods

### Bacterial strain and culture conditions

*Halomonas malpeensis* YU-PRIM-29^T^ was originally isolated from the rhizosphere soil in the coastal region of Malpe (13° 21′ 10.22″ N, 74° 42′ 29.99″ E). It was cultured in Zobell marine agar 2216 (HiMedia, India) at 32 °C. For DNA isolation, the bacterium was cultured in Zobell marine broth 2216 by incubating at 32 °C, under shaking (120 rpm) for 24 h. The cells were harvested by centrifugation (7500 rpm), washed and lysed by lysis buffer followed by proteinase K treatment. The DNA was extracted using Qiagen kit (Cat No./ID: 51304) following the manufacturer’s instruction for Gram-negative bacteria. The DNA concentration and purity were assessed by the absorbance readings at 260 to 280 nm using a Nanodrop spectrophotometer (Colibri, Titertek Berthold). Polymerase Chain Reaction (PCR) was performed to amplify the 16S rRNA region, and the quality of the DNA sample was checked by gel electrophoresis prior to genome sequencing.

### Sequencing, assembly and annotation

Illumina sequencing was done on a MiSeq platform using MiSeq Reagent Kit v3, 600 Cycles reagents (Catalog # MS-102-3003). It produced 1,173,355 raw reads after pre-processing (adaptor trimming) for each of R1 and R2. This gave average genome coverage of 190X for a 5 Mb genome size with a data yield of 1,598,696 reads. FASTQC was used to assess the raw reads quality and trimming was performed by trimmomatic (Version 0.35), default settings, identifying a Phred cutoff of Q20. The sequence was uploaded to the web annotation service RAST (http://rast.nmpdr.org/rast.cgi)^62^ as well as PATRIC (https://www.patricbrc.org/) for automated annotation.

De novo assembly of the sequences were performed using SPAdes 3.11.1 and the resulting assembly with best N50 value was taken into gene prediction using Prokka (kbase.us)^63^. The PATRIC gene features were considered as a basis for annotation. PATRIC output was checked and corroborated by comparing to that from Prokka and RAST. The construction of genomic and metabolic pathways was executed using all three. These data sources were combined to affirm product description for predicted proteins. A total of 11 *Halomonas* genomes were used for comparing protein-family across genomic groups (10 from PATRIC and the new genome from this study) using Protein Family Sorter tool in PATRIC. The gene features of essential biosystems were further manually confirmed using BLASTp (https://blast.ncbi.nlm.nih.gov/Blast.cgi) against non-redundant database of NCBI (details provided in Supplementary Table S9). The proteins involved in EPS and pigment production pathways were identified by the local tBLASTn of selected EPS/pigment production pathway proteins from known microbial genomes/proteomes against whole genome sequence with alignment length of at least 80% and e-value cut-off of ≤ 10^−5^ (details provided in Supplementary Table S10). The best BLAST hit with the highest alignment length percentage and identity match was assigned as the annotation of the predicted gene. Essential enzyme functional prediction was obtained from KEGG (http://www.genome.jp/kegg/)^64^ using KAAS server^65^. This functional annotation was used to reconstruct the metabolic pathways related to EPS biosynthesis and pigment production. The dbCAN2 meta server (http://cys.bios.niu.edu/dbCAN2/index.php) was used to identify the glycosyltransferases involved in EPS biosynthesis. Similarity searches against Transporter Classification Database (TCDB) (www.tcdb.org) was performed to confirm the genes coding for the ABC transporters involved in EPS export and annotations of best-matching hits with an e-value cut-off of 10^−9^. WebMGA was used for the functional characterisation of the protein coding genes^66^ and were mapped to the COG functional category assignment^67^. For identifying the functional features of the proteins InterPro^68^ and UniProt BLAST^69^ were used. Biosynthetic gene clusters for secondary metabolites were predicted using antiSMASH^70^ with default search parameters. Based on the genes identified, visualisation of the biosynthesis pathways for EPS production and transport as well as for the pigment were done using CorelDraw Technical Suite, 2019 and GraphPad Prism 5.03 software.

### Isolation of EPS, pigment and other metabolites from *H. malpeensis* cultures

For extracting the EPS, bacteria were cultured in Marine broth for 72 h under shaking at 32 °C. EPS was isolated from the cell-free supernatant by cold ethanol precipitation. The harvested EPS was dialysed against MilliQ water, lyophilised and the yield was recorded. The basic biochemical characterisation of the EPS was performed by estimating the total sugar and total protein content by phenol sulphuric acid^71^ and Bradford methods^72^ respectively. Basic structural characterisation of the EPS was performed by the FTIR spectroscopy.

For estimating the growth and EPS yield, bacteria were grown in MY media (g/L; sodium chloride 51.3, magnesium sulphate heptahydrate 13.0, yeast extract 3.0, magnesium chloride 9.0, potassium chloride 1.3, sodium bicarbonate 0.05, peptone 5.0, glucose 10, malt extract 3.0, calcium chloride 0.2, sodium bromide 0.15, and ferrous chloride tetrahydrate 0.036) containing 7.5% salt supplemented with different carbon sources (glucose, fructose, sucrose, maltose, arabinose, xylose and mannose). The culture conditions included temperature 32 ℃, pH 7.2, aeration 1:5 and agitation 120 rpm. Growth was monitored based on the OD_600_ readings and EPS was harvested by chilled ethanol precipitation. EPS was purified by dialysis using MilliQ water and lyophilised to estimate the dry weight.

For the extraction of the yellow pigment, the cell pellet collected by centrifugation was used. Pigment was extracted several times with methanol till the cells were bleached completely. The extracts were pooled and concentrated using a vacuum evaporator. To this hexane and distilled water were added and mixed well to separate the other organics from the pigment. The pigment extracted in the hexane layer was subjected to UV–Vis and fluorescent spectrophotometry against standard zeaxanthin. Further, confirmation of zeaxanthin was made based on the LC–MS/MS analysis (detailed methods are given in Supplementary_ Methods file). The isolated pigment was tested for antioxidant activities by DPPH scavenging^73^, nitric oxide scavenging^74^, hydroxyl radical scavenging^75^ and lipid peroxidation inhibition activities^76^ for which the IC_50_ values were calculated.

The biofilm forming ability of the bacteria was tested using crystal violet staining method^51^. For this, bacteria were inoculated to Zobell marine broth in polystyrene cuvettes and incubated for different time intervals up to 96 h. The planktonic cells were removed by carefully decanting the contents and the static biofilm was washed with sterile PBS twice, fixed with methanol (10 min), washed again and dried. After drying, 0.1% crystal violet was added and kept for staining (5 min). The stain was solubilised using acetic acid (33%) and the absorbance was recorded at 580 nm. The biofilm adherence capacity was based on the OD_580_ readings compared with the blank as OD ≤ ODc (non-adherent), OD < ODc ≤ 2 × ODc (weakly adherent), 2 × ODc < OD ≤ 4 × ODc assessed (moderately adherent) and 4 × ODc < OD (strongly adherent)^51^. For FESEM, the biofilm was developed on a sterile glass coupon (1 × 1 cm), fixed with methanol, dehydrated and subjected to sputter coating prior to FESEM analysis^77^.

The PHA producing ability of the bacterium was tested by Sudan Black B staining method. Here, the bacterial colonies in the agar plates were stained with 0.02% of ethanolic solution of Sudan Black B dye for 1 h. The excess stain was removed by 70% ethanol. The darkly stained culture plate was photographed. For microscopy, bacterial smear was prepared by heat fixing, stained with Sudan Black B. Xylene was used for decolourizing and the cells were counter stained using 0.05% safranin for 10 s. The stained cells were observed under  100X magnification^78^. Extraction of PHA was performed from the bacterial cells after lyophilisation according to previously described methods^79^. The PHA content was determined as the percent ratio of PHA to cell dry weight. FTIR spectrum of PHA was recorded using Shimadzu FTIR spectrophotometer (4000–400 cm^−1^, spectral resolution of 4 cm^−1^ and 45 scans). The spectrum obtained was plotted using Origin 2017 SR2 software.

For extraction of ectoine a previously described method was used^80^. Briefly, ectoine was extracted from the cell pellet with methanol/chloroform/water (10/5/4 v/v/v) by vigorous shaking for 90 min. Equal volume of chloroform and water (130 µL/mL) was added, mixed well for 30 min and collected from the aqueous phase by centrifugation (6500 rpm, 30 min). Ectoine was identified using LC–MS/MS against the standard (detailed methodology in Supplementary document).

#### Bacterium strain and sequence

The sequencing data of the draft genome of halophilic *H. malpeensis* YU-PRIM-29^T^ is available online as BioProject PRJNA579246, NCBI taxonomy ID 1172368 from the NCBI database. The genome description and the predictive annotation are available in PATRIC server with genome ID 2745.436 and RAST server with genome ID 2745.437. The Whole Genome Shotgun project of strain YU-PRIM-29^T^ was deposited at DDBJ/EMBL/GenBank under the accession number WHVL00000000. The version described in this paper is version WHVL00000000. The 16S rRNA gene sequence is available in GenBank with the accession ID JQ730736.

## Supplementary Information

Supplementary Information 1.

Supplementary Information 2.

## Data Availability

The datasets generated during and/or analysed during this study are obtainable from the corresponding author on reasonable request.
