# Supplementary material for: Genome analysis of a halophilic bacterium Halomonas malpeensis YU-PRIM-29T reveals its exopolysaccharide and pigment producing capabilities
Source: Sci Rep. 2021 Jan 18;11:1749. doi: 10.1038/s41598-021-81395-1 (PMC7814019; doi:10.1038/s41598-021-81395-1)
Supplement: Supplementary file 1 — Supplementary Information 1. [file 41598_2021_81395_MOESM1_ESM.pdf]

Supplementary information for

Genome analysis of a halophilic bacterium *Halomonas malpeensis* YU-PRIM-29^T reveals its exopolysaccharide and pigment producing capabilities

Athmika[#] Sudeep D Ghate[#] Arun AB, Sneha S Rao, ST Arun Kumar, Mrudula Kinarulla Kandiyil, Kanekar Saptami and Rekha P D[*]

Yenepoya Research Centre, Yenepoya (Deemed to be University), University Road,

Deralakatte, Mangalore 575018, India

#Equal contribution, *Corresponding author

E-mail address: rekhapd@hotmail.com, dydirectoryrc@yenepoya.edu.in (Rekha P.D.

Supplementary File Information for Sample 1 (Ectoin sample) of Ectoin sample.wiff
File Name: Ectoin sample_2.wiff
File Path: D:\Analyst
Data\Projects\Ection_Positive_MRM\2020_09_24\Data\Ectoin sample_09282020\
Original Name: Ectoin sample_09282020\Ectoin sample_2.wiff
Software Version: Analyst 1.6.3

Log Information from Devices at Start of acquisition:
Software Application Analyst Device Driver 0

Time from start =0.0000 min
Analyst Device Driver User=\ Computer=6500QTRAP-PC
Pump1 G7120A Binary Pump
Mixer Type Jet Weaver V100 Mixer
Connection type IP Address
IP Address 192.168.254.11
Solvent Selection Valve Installed Yes
Configured Pressure Unit bar
ISET installed No
Firmware Revision B.07.25 [0013]
Serialnumber DEBA200299
Moduletype G7120A

Time from start =0.0167 min
Analyst Device Driver User=\ Computer=6500QTRAP-PC
Sampler1 G7167B Multisampler
Metering Type G4267-60042
Metering Name 40 µL Analytical Head
Metering Volume 40.0
Diameter 2.000
Right Seat Capillary Type G4267-87012
Right Seat Cap. Name Seat assembly 0.12 mm 1290 Infinity LC
Right Seat Cap. Injection Volume 0.0
Right Seat Cap. Physical Volume 1.5
Right Loop Capillary Type G4267-60300
Right Loop Cap. Name Sample Loop-Flex 20 µL right
Right Loop Cap. Injection Volume 20.0
Right Loop Cap. Physical Volume 33.0
Right Needle Max. Injection Volume 20.00
Ref. Vial Array Type G4267-40071
Ref. Vial Array Name Reference vial rack (5)
Moduletype
Serialnumber
Connection type IP Address
IP Address
192.168.254.11
Cooler Installed Yes

Temperature control mode Constant temperature mode (control setting)
Multi-wash Installed No
Dual-needle Installed No
Firmware Revision D.07.25 [0013]
Serialnumber DEBAQ00586
Moduletype G7167B

Mass Spectrometer QTRAP 6500 Low Mass 0
Config Table Version 02
Firmware Version ------- ------- PIL1602 PIB1100
Component Name LINEAR ION TRAP QUADRUPOLE LC/MS/MS MASS SPECTROMETER
Component ID QTRAP 6500
Manufacturer AB SCIEX INSTRUMENTS
Model 5035182-K
Serial Number BL210551508

Analyst Device Driver User=\ Computer=6500QTRAP-PC
Column Comp.1 G7116B Column Comp.
Connection type IP Address
IP Address 192.168.254.11
Valve Installed No
Left Tag Reader Installed No
Right Tag Reader Installed No
Firmware Revision D.07.25 [0013]
Slave Firmware C.07.21 [0001]
Serialnumber DEBAZ00370
Moduletype G7116B

Time from start =0.0500 min
Analyst Device Driver User=\ Computer=6500QTRAP-PC
Injection Volume used 10 µl

Time from start =0.0667 min
Mass Spectrometer QTRAP 6500 Low Mass 0
Start of Run - Detailed Status
Vacuum Status At Pressure
Vacuum Gauge (10e-5 Torr) 2.4
Backing Pump Ok
Interface Pump Bad
Curtain Gas Bad
Interface Turbo Pump Normal
Analyzer Turbo Pump Off
Sample Introduction Status Ready

Source/Ion Path Electronics	On
Source Type	Turbo Spray IonDrive
Source Temperature (at setpoint)	450.0 C
Source Exhaust Pump	On
Injection Manifold	Bypass

Time from start =0.6167 min

Mass Spectrometer QTRAP 6500 Low Mass 0

End of Run - Detailed Status

Vacuum Status	At Pressure
Vacuum Gauge (10e-5 Torr)	2.3
Backing Pump	Ok
Interface Pump	Bad
Curtain Gas	Bad
Interface Turbo Pump	Normal
Analyzer Turbo Pump	Off
Sample Introduction Status	Ready
Source/Ion Path Electronics	On
Source Type	Turbo Spray IonDrive
Source Temperature (at setpoint)	449.0 C
Source Exhaust Pump	On
Injection Manifold	Bypass

Time from start =10.6333 min

Acquisition Info

Acquisition Method: \Ecotin_ISO_25092020.dam
Acquisition Path: D:\Analyst Data\Projects\Ection_Positive_MRM\2020_09_24\Acquisition Methods\
First Sample Started: 28 September 2020 13:02:03
Last Sample Finished: 28 September 2020 13:02:03
Sample Acq Time: 28 September 2020 13:02:03
Sample Acq Duration: 10min0sec
Number of Scans: 0
Periods in File: 1
Batch Name: \New Batch.dab
Batch Path: D:\Analyst Data\Projects\Ection_Positive_MRM\2020_09_24\Batch\
Submitted by: 6500Qtrap-PC\abservice(abservice)
Logged-on User: abservice
Synchronization Mode: Manual/AAO Sync
Auto-Equilibration: Off
Comment:
Software Version: Analyst 1.6.3
Set Name: Ectoin sample_09282020
Sample Name Ectoin sample_2
Sample ID
Sample Comments:
Autosampler Vial: 75
Rack Code: Multi Drawer

Rack Position:	1
Plate Code:	*96Agilent*
Plate Position	5

Software Application Properties

Display Name:	Analyst Device Driver
Identifier Key:	{CA8AA7DB-31CA-4FFB-AA8E-E938DB87364E}
Method Filename:	None

Method Data:

Pump1 (G7120A): "Binary Pump"

Automatic Stroke Calculation A:	Yes
Stoptime Mode:	No limit
Posttime Mode:	Off
Flow:	0.300
Use Solvent Types:	Yes
Stroke Mode:	Synchronized
Low Pressure Limit:	0.00
High Pressure Limit:	1300.00
Max. Flow Ramp Up:	100.000
Max. Flow Ramp Down:	100.000
Expected Mixer:	No check
Channel:	A
Ch. 1 Solv.:	100.0 % Water V.03
Name 1:	
Ch2 Solv.:	100.0 % Water V.03
Name 2:	
Selected:	Ch. 1
Used:	Yes
Percent:	50.00
Channel:	B
Ch. 1 Solv.:	100.0 % Acetonitrile V.03
Name 1:	
Ch2 Solv.:	100.0 % Acetonitrile V.03
Name 2:	
Selected:	Ch. 1
Used:	Yes
Percent:	50.00
Time:	10.00
A:	50.00
B:	50.00
Flow:	0.300
Pressure:	1300.00

Sampler1 (G7167B): "Multisampler"

Draw Speed:	100.0

Eject Speed: 400.0
Wait Time After Drawing: 1.2
Needle Wash Mode: Flush Port
Duration: 3
Needle Wash Mode: Standard Wash
Injection Volume: 10.00
Overlap Injection Enabled: No
Injection Valve to Bypass for Delay Volume Reduction:
Sample Flush-Out Factor: 5.0
Draw Position Offset: 1.0
Use Vial/Well Bottom Sensing: Yes
Stoptime Mode: No Limit
Posttime Mode: Off

Column Comp.1 (G7116B): "Column Comp."
Enable Analysis Left Temperature On: Yes
Enable Analysis Left Temperature Value: 0.8
Left Temp. Equilibration Time: 0.0
Temperature Control Mode:
Temperature Set
Temperature: 35.0
Enable Analysis Right Temperature On: Yes
Enable Analysis Right Temperature Value: 0.8
Right Temp. Equilibration Time: 0.0
Right temperature Control Mode:
Temperature Set
Right temperature: 35.0
Enforce column for run enabled: No
Stoptime Mode: As pump/injector
Posttime Mode: Off
Ready when front door open: No
Position Switch After Run: Do not switch

Quantitation Information:
Sample Type: Unknown
Dilution Factor: 1.000000

Custom Data:

Quantitation Table:

Period 1:

Scans in Period: 33293
Relative Start Time: 0.00 msec
Experiments in Period: 1

Period 1 Experiment 1:

Scan Type:	MRM (MRM)
Scheduled MRM:	No
Polarity:	Positive
Scan Mode:	N/A
Resolution Q1:	Unit
Resolution Q3:	Unit
Intensity Thres.:	0.00 cps
Settling Time:	20.0000 msec
MR Pause:	5.0070 msec
MCA:	No
Step Size:	0.00 Da

Q1 Mass (Da)	Q3 Mass (Da)	Dwell(msec)	Param	Start	Stop
143.000	68.000	1.00	DP	52.00	52.00
			EP	6.70	6.70
			CE	29.04	29.04
			CXP	32.00	32.00

Q1 Mass (Da)	Q3 Mass (Da)	Dwell(msec)	Param	Start	Stop
143.000	82.900	1.00	DP	53.00	53.00
			EP	8.80	8.80
			CE	39.00	39.00
			CXP	39.00	39.00

Q1 Mass (Da)	Q3 Mass (Da)	Dwell(msec)	Param	Start	Stop
143.000	97.000	1.00	DP	48.00	48.00
			EP	5.30	5.30
			CE	23.00	23.00
			CXP	38.00	38.00

Parameter Table (Period 1 Experiment 1)

CUR:	30.00
CAD:	High
TEM:	450.00
GS1:	35.00
GS2:	35.00
IS:	4500.00

Resolution tables

Quad 1 Positive Unit Scan Speed = 10 Da/s

Last Modification Date Time: January 14, 2020 10:10:26

IE1 1.000

Mass (Da)	Offset Value
59.050	-0.010
175.133	-0.054
500.380	-0.200
616.464	-0.256
906.673	-0.400

Quad 3 Positive Unit Scan Speed = 10 Da/s
Last Modification Date Time: January 14, 2020 09:43:59

IE3 1.700

Mass (Da)	Offset Value
59.050	0.060
175.133	0.120
500.380	0.308
616.464	0.370
906.673	0.490

Calibration tables
Quad 1 Positive Unit Resolution Scan Speed = 10 Da/s
Last Modification Date Time: January 14, 2020 10:09:46

Mass (Da)	Dac Value
59.050	10636
175.133	31931
500.380	91649
616.464	112964
906.673	166253

Quad 3 Positive Unit Resolution Scan Speed = 10 Da/s
Last Modification Date Time: January 14, 2020 10:12:20

Mass (Da)	Dac Value
59.050	10578
175.133	31769
500.380	91209
616.464	112422
906.673	165460

Instrument Parameters:
Detector Parameters (Positive):
CEM 1500.0

Keyed Text:
File was created with the software version: Analyst 1.6.3

File Information for Sample 1 (Zeaxanthin_Extract_2) of Zeaxanthin_Extract_2.wiff
File Name: Zeaxanthin_Extract_2.wiff
File Path: D:\Analyst Data\Projects\Ection_Positive_MRM\2020_09_24\Data\Zeaxanthin_Extract_09282020\
Original Name: Zeaxanthin_Extract_09282020\Zeaxanthin_Extract_2.wiff
Software Version: Analyst 1.6.3

Log Information from Devices at Start of acquisition:
Software Application Analyst Device Driver 0

Time from start =0.0000 min
Analyst Device Driver User=\ Computer=6500QTRAP-PC
Pump1 G7120A Binary Pump
Mixer Type Jet Weaver V100 Mixer
Connection type IP Address
IP Address 192.168.254.11
Solvent Selection Valve Installed Yes
Configured Pressure Unit bar
ISET installed No
Firmware Revision B.07.25 [0013]
Serialnumber DEBA200299
Moduletype G7120A

Time from start =0.0167 min
Analyst Device Driver User=\ Computer=6500QTRAP-PC
Sampler1 G7167B Multisampler
Metering Type G4267-60042
Metering Name 40 µL Analytical Head
Metering Volume 40.0
Diameter 2.000
Right Seat Capillary Type G4267-87012
Right Seat Cap. Name Seat assembly 0.12 mm 1290 Infinity LC
Right Seat Cap. Injection Volume 0.0
Right Seat Cap. Physical Volume 1.5
Right Loop Capillary Type G4267-60300
Right Loop Cap. Name Sample Loop-Flex 20 µL right
Right Loop Cap. Injection Volume 20.0
Right Loop Cap. Physical Volume 33.0
Right Needle Max. Injection Volume 20.00
Ref. Vial Array Type G4267-40071
Ref. Vial Array Name Reference vial rack (5)
Moduletype
Serialnumber
Connection type IP Address
IP Address
192.168.254.11
Cooler Installed Yes

Temperature control mode Constant temperature mode (control setting)
Multi-wash Installed No
Dual-needle Installed No
Firmware Revision D.07.25 [0013]
Serialnumber DEBAQ00586
Moduletype G7167B

Mass Spectrometer QTRAP 6500 Low Mass 0
Config Table Version 02
Firmware Version ------- ------- PIL1602 PIB1100
Component Name LINEAR ION TRAP QUADRUPOLE LC/MS/MS MASS SPECTROMETER
Component ID QTRAP 6500
Manufacturer AB SCIEX INSTRUMENTS
Model 5035182-K
Serial Number BL210551508

Analyst Device Driver User=\ Computer=6500QTRAP-PC
Column Comp.1 G7116B Column Comp.
Connection type IP Address
IP Address 192.168.254.11
Valve Installed No
Left Tag Reader Installed No
Right Tag Reader Installed No
Firmware Revision D.07.25 [0013]
Slave Firmware C.07.21 [0001]
Serialnumber DEBAZ00370
Moduletype G7116B

Time from start =0.0500 min
Analyst Device Driver User=\ Computer=6500QTRAP-PC
Injection Volume used 10 µl

Time from start =0.0667 min
Mass Spectrometer QTRAP 6500 Low Mass 0
Start of Run - Detailed Status
Vacuum Status At Pressure
Vacuum Gauge (10e-5 Torr) 2.3
Backing Pump Ok
Interface Pump Bad
Curtain Gas Bad
Interface Turbo Pump Normal
Analyzer Turbo Pump Off
Sample Introduction Status Ready

Source/Ion Path Electronics	On
Source Type	Turbo Spray IonDrive
Source Temperature (at setpoint)	449.0 C
Source Exhaust Pump	On
Injection Manifold	Bypass

Time from start =0.6167 min

Mass Spectrometer QTRAP 6500 Low Mass 0

End of Run - Detailed Status

Vacuum Status	At Pressure
Vacuum Gauge (10e-5 Torr)	2.3
Backing Pump	Ok
Interface Pump	Bad
Curtain Gas	Bad
Interface Turbo Pump	Normal
Analyzer Turbo Pump	Off
Sample Introduction Status Ready	
Source/Ion Path Electronics	On
Source Type	Turbo Spray IonDrive
Source Temperature (at setpoint)	450.0 C
Source Exhaust Pump	On
Injection Manifold	Bypass

Time from start =12.6167 min

Acquisition Info

Acquisition Method:	\Zeaxanthin_Pos_MRM_LC_09242020.dam
Acquisition Path:	D:\Analyst Data\Projects\Ection_Positive_MRM\2020_09_24\Acquisition Methods\
First Sample Started:	28 September 2020 21:02:23
Last Sample Finished:	28 September 2020 21:02:23
Sample Acq Time:	28 September 2020 21:02:23
Sample Acq Duration:	11min60sec
Number of Scans:	0
Periods in File:	1
Batch Name:	\New Batch.dab
Batch Path:	D:\Analyst Data\Projects\Ection_Positive_MRM\2020_09_24\Batch\
Submitted by:	6500Qtrap-PC\abservice(abservice)
Logged-on User:	abservice
Synchronization Mode:	Manual/AAO Sync
Auto-Equilibration:	Off
Comment:	
Software Version:	Analyst 1.6.3
Set Name:	Zeaxanthin_Extract_09282020
Sample Name	Zeaxanthin_Extract_2
Sample ID	
Sample Comments:	
Autosampler Vial:	77
Rack Code:	Multi Drawer

Rack Position: 1
Plate Code: *96Agilent*
Plate Position 5

Software Application Properties

Display Name: Analyst Device Driver
Identifier Key: {CA8AA7DB-31CA-4FFB-AA8E-E938DB87364E}
Method Filename: None

Method Data:

Pump1 (G7120A): "Binary Pump"
Automatic Stroke Calculation A: Yes
Stoptime Mode: No limit
Posttime Mode: Off
Flow: 0.300
Use Solvent Types: Yes
Stroke Mode: Synchronized
Low Pressure Limit: 0.00
High Pressure Limit: 1300.00
Max. Flow Ramp Up: 100.000
Max. Flow Ramp Down: 100.000
Expected Mixer: No check
Channel: A
Ch. 1 Solv.: 100.0 % Water V.03
Name 1:
Ch2 Solv.: 100.0 % Water V.03
Name 2:
Selected: Ch. 1
Used: Yes
Percent: 98.00
Channel: B
Ch. 1 Solv.: 100.0 % Acetonitrile V.03
Name 1:
Ch2 Solv.: 100.0 % Acetonitrile V.03
Name 2:
Selected: Ch. 1
Used: Yes
Percent: 2.00
Time: 1.00
A: 98.00
B: 2.00
Flow: 0.300
Pressure: ---
Time: 2.00
A: 70.00
B: 30.00

Flow:	0.300
Pressure:	---
Time:	5.00
A:	40.00
B:	60.00
Flow:	0.300
Pressure:	---
Time:	6.00
A:	2.00
B:	98.00
Flow:	0.300
Pressure:	---
Time:	8.00
A:	2.00
B:	98.00
Flow:	0.300
Pressure:	---
Time:	9.00
A:	98.00
B:	2.00
Flow:	0.300
Pressure:	---
Time:	10.00
A:	98.00
B:	2.00
Flow:	0.300
Pressure:	---

Sampler1 (G7167B): "Multisampler"

Draw Speed:	100.0
Eject Speed:	400.0
Wait Time After Drawing:	1.2
Needle Wash Mode:	Flush Port
Duration:	3
Needle Wash Mode:	Standard Wash
Injection Volume:	10.00
Overlap Injection Enabled:	No
Injection Valve to Bypass for Delay Volume Reduction:	
Sample Flush-Out Factor:	5.0
Draw Position Offset:	0.0
Use Vial/Well Bottom Sensing:	No
Stoptime Mode:	No Limit
Posttime Mode:	Off

Column Comp.1 (G7116B): "Column Comp."

Enable Analysis Left Temperature On:	Yes
Enable Analysis Left Temperature Value:	0.8
Left Temp. Equilibration Time:	0.0
Temperature Control Mode:	
Temperature Set	

Temperature:	40.0	
Enable Analysis Right Temperature On:		Yes
Enable Analysis Right Temperature Value:		0.8
Right Temp. Equilibration Time:		0.0
Right temperature Control Mode:		
Temperature Set		
Right temperature:	40.0	
Enforce column for run enabled:		No
Stoptime Mode:	As pump/injector	
Posttime Mode:	Off	
Ready when front door open:		No
Position Switch After Run:	Do not switch	

Quantitation Information:

Sample Type:	Unknown
Dilution Factor:	1.000000

Custom Data:

Quantitation Table:

Period 1:

Scans in Period:	9595
Relative Start Time:	0.00 msec
Experiments in Period:	1

Period 1 Experiment 1:

Scan Type:	MRM (MRM)
Scheduled MRM:	No
Polarity:	Positive
Scan Mode:	N/A
Resolution Q1:	Unit
Resolution Q3:	Unit
Intensity Thres.:	0.00 cps
Settling Time:	20.0000 msec
MR Pause:	5.0070 msec
MCA:	No
Step Size:	0.00 Da

Q1 Mass (Da)	Q3 Mass (Da)	Dwell(msec)	Param	Start	Stop
569.900	398.800	10.00	DP	86.00	86.00
			CE	39.00	39.00
			CXP	44.00	44.00

Q1 Mass (Da)	Q3 Mass (Da)	Dwell(msec)	Param	Start	Stop
569.900	102.200	10.00	DP	76.00	76.00
			CE	63.00	63.00
			CXP	18.00	18.00

Q1 Mass (Da)	Q3 Mass (Da)	Dwell(msec)	Param	Start	Stop
569.900	323.300	10.00	DP	16.00	16.00
			CE	29.00	29.00
			CXP	40.00	40.00

Q1 Mass (Da)	Q3 Mass (Da)	Dwell(msec)	Param	Start	Stop
569.900	310.700	10.00	DP	51.00	51.00
			CE	39.00	39.00
			CXP	16.00	16.00

Q1 Mass (Da)	Q3 Mass (Da)	Dwell(msec)	Param	Start	Stop
569.900	255.100	10.00	DP	76.00	76.00
			CE	41.00	41.00
			CXP	30.00	30.00

Parameter Table (Period 1 Experiment 1)

CUR:	30.00
CAD:	High
TEM:	450.00
GS1:	35.00
GS2:	35.00
IS:	4500.00
EP	9.00

Resolution tables

Quad 1 Positive Unit Scan Speed = 10 Da/s

Last Modification Date Time: January 14, 2020 10:10:26

IE1 1.000

Mass (Da)	Offset Value
59.050	-0.010
175.133	-0.054
500.380	-0.200
616.464	-0.256
906.673	-0.400

Quad 3	Positive	Unit	Scan Speed = 10 Da/s

Last Modification Date Time: January 14, 2020 09:43:59

IE3	1.700
Mass (Da)	Offset Value
59.050	0.060
175.133	0.120
500.380	0.308
616.464	0.370
906.673	0.490

Calibration tables

Quad 1	Positive	Unit Resolution	Scan Speed = 10 Da/s

Last Modification Date Time: January 14, 2020 10:09:46

Mass (Da)	Dac Value
59.050	10636
175.133	31931
500.380	91649
616.464	112964
906.673	166253

Quad 3	Positive	Unit Resolution	Scan Speed = 10 Da/s

Last Modification Date Time: January 14, 2020 10:12:20

Mass (Da)	Dac Value
59.050	10578
175.133	31769
500.380	91209
616.464	112422
906.673	165460

Instrument Parameters:

Detector Parameters (Positive):

CEM	1500.0

Keyed Text:

File was created with the software version: Analyst 1.6.3
